# Peroxiredoxin II with dermal mesenchymal stem cells accelerates wound healing

**DOI:** 10.18632/aging.202990

**Published:** 2021-05-24

**Authors:** Mei-Hua Jin, Nan-Nan Yu, Ying-Hua Jin, Ying-Ying Mao, Lin Feng, Yue Liu, Ai-Guo Wang, Hu-Nan Sun, Taeho Kwon, Ying-Hao Han

**Affiliations:** 1 College of Life Science and Technology, Heilongjiang Bayi Agricultural University, Daqing 163319, Heilongjiang, P.R. China; 2 Department of Plasma Bioscience and Display, Plasma Bioscience Research Center, Applied Plasma Medicine Center, Kwangwoon University, Nowon-gu 01897, Seoul, Republic of Korea; 3 Library and Information Center, College of Life Science and Technology, Heilongjiang Bayi Agricultural University, Daqing 163319, Heilongjiang, P.R. China; 4 Laboratory Animal Center, Dalian Medical University, Dalian 116044, Liaoning, P.R. China; 5 Primate Resources Center, Korea Research Institute of Bioscience and Biotechnology (KRIBB), Jeongeup-si 56216, Jeonbuk, Republic of Korea

**Keywords:** peroxiredoxin II, dermal mesenchymal stem cells, vascular endothelial growth factor (VEGF), reactive oxygen species, microRNA

## Abstract

Peroxiredoxin II (Prx II) is involved in proliferation, differentiation, and aging in various cell types. However, Prx II-mediated stem cell regulation is poorly understood. Here, dermal mesenchymal stem cells (DMSCs), cell-growth factor-rich conditioned medium from DMSCs (DMSC-CM), and DMSC-derived exosomes (DMSC-Exos) were used to explore the regulatory role of Prx II in DMSC wound healing. Following treatment, wound healing was significantly decelerated in Prx II^−/−^ DMSCs than in Prx II^+/+^ DMSCs. *In vitro* stimulation with 10 μM H_2_O_2_ significantly increased apoptosis in Prx II^−/−^ DMSCs compared with Prx II^+/+^ DMSCs. The mRNA expression levels of EGF, b-FGF, PDGF-B, and VEGF did not significantly differ between Prx II^−/−^ and Prx II^+/+^ DMSCs. Fibroblasts proliferated comparably when treated with Prx II^+/+^ DMSC-CM or Prx II^−/−^ DMSC-CM. Wound healing was significantly higher in the Prx II^−/−^ DMSC-Exos-treated group than in the Prx II^+/+^ DMSCs-Exos-treated group. Moreover, microRNA (miR)-21-5p expression levels were lower and miR-221 levels were higher in Prx II^−/−^ DMSCs than in Prx II^+/+^ DMSCs. Therefore, our results indicate that Prx II accelerated wound healing by protecting DMSCs from reactive oxygen species-induced apoptosis; however, Prx II did not regulate cell/growth factor secretion. Prx II potentially regulates exosome functions via miR-21-5p and miR-221.

## INTRODUCTION

Skin wounds comprise one of the most common clinical conditions and are primarily caused by surgery, traffic accidents, fires, and certain metabolic diseases [1]. However, if a severe wound cannot heal in time, it may cause local infection, motor dysfunction, and even death; thus, skin wounds may represent severe health hazards. However, the optimal treatment strategy for skin wounds requires multiple cell types and complex signaling pathways. Healing of skin wounds is a substantial and complex biological process involving cell migration, proliferation, angiogenesis, and extracellular matrix deposition [2, 3]. Unfortunately, current surgical and pharmacotherapeutic strategies cannot accomplish the aforementioned requirements. In addition, numerous reports have shown that mesenchymal stem cells (MSCs) can participate in the biological processes associated with wound healing and play active roles owing to their unique characteristics [4]. However, a comprehensive understanding of the mechanism underlying the role of MSCs in promoting wound healing has not been attained, which has severely reduced the clinical applications of stem cell therapy.

Previous findings showed that stem cell-based treatment promoted skin wound healing and that antioxidant proteins such as catalase and MnSOD played important roles [5, 6]. Increased expression levels of antioxidant enzymes in stem cells can help stem cells resist oxidative stress, thereby improving their therapeutic potential. Furthermore, peroxiredoxin II (Prx II) is an antioxidant enzyme that can promptly quench intracellular reactive oxygen species (ROS) at low concentrations [7]. We previously reported that the Wnt/β-catenin signaling pathway was aberrantly activated and the cell cycle was arrested in Prx II-knockout dermal mesenchymal stem cells (DMSCs) [8]. DMSCs, located in the skin, differentiate into fibroblasts and secrete active substances enabling them to participate in would healing in normal skin tissue [9]. Therefore, we hypothesized that Prx II potentially contributes to the efficacy of DMSCs in treating skin wounds.

Stem cells have been reported to promote wound healing by proliferating and differentiating into various cells needed for wound healing, replacing damaged cells, and filling wound sites [10, 11]. Furthermore, stem cells also secrete numerous bioactive substances including cell-growth factors and exosomes, thus promoting proliferation and the physiological functions of various cells necessary for wound healing [12, 13]. Therefore, to comprehensively and systematically investigate the regulatory role of Prx II in the treatment of skin wound healing using DMSCs, we employed Prx II^+/+^ and Prx II^−/−^ DMSCs. Importantly, cell-growth factor-rich conditioned medium (Prx II^+/+^ DMSCs-CM and Prx II^−/−^ DMSCs-CM) and exosomes (Prx II^+/+^ DMSC-Exos and Prx II^−/−^ DMSC-Exos) were used to treat skin wounds in mice. In this study, we compared the outcomes of cell therapy, cell-growth factor therapy, and exosome therapy in Prx II-deficient skin tissues during wound healing. Through *in vitro* experiments, we briefly investigated the mechanism of action of Prx II during stem cell-based treatment of skin wounds.

## RESULTS

### Characterization of DMSCs

**Figure 1:**
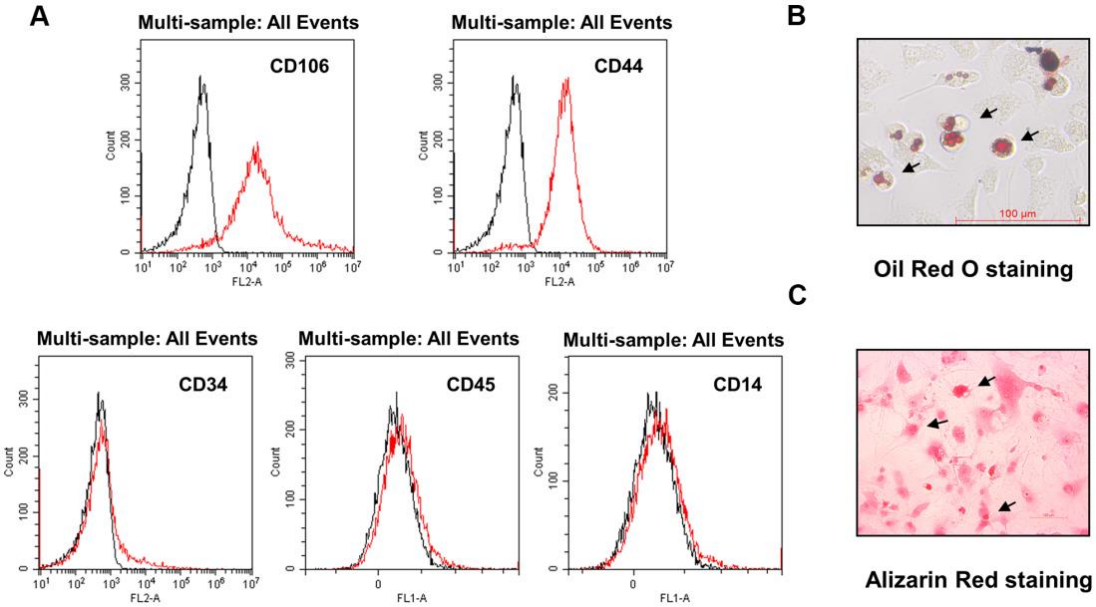
**Characterization of DMSCs.** (**A**) DMSCs were analyzed by FACS after staining with FITC- or PE-conjugated control isotype IgG (black peaks) or antibodies against the indicated cell-surface proteins. (**B**) DMSC differentiation. DMSCs were cultured in appropriate differentiation media to promote differentiation into adipocytes, as indicated by oil red O staining, and (**C**) osteoblasts, as indicated by alizarin red staining.

Fluorescence-activated cell sorting (FACS) analysis of DMSCs at passage four revealed that most cells were negative for CD34, CD45, and CD14, but strongly expressed MSC-specific surface antigens such as CD44 and CD106 (Figure 1A). When cultured in differentiation medium, the isolated DMSCs could differentiate into adipocytes (Figure 1B) and osteoblasts (Figure 1C), as demonstrated through oil red O and alizarin red staining, respectively, indicating that DMSCs were successfully extracted.

### Deletion of Prx II inhibited DMSC-based treatment of skin wounds

**Figure 2:**
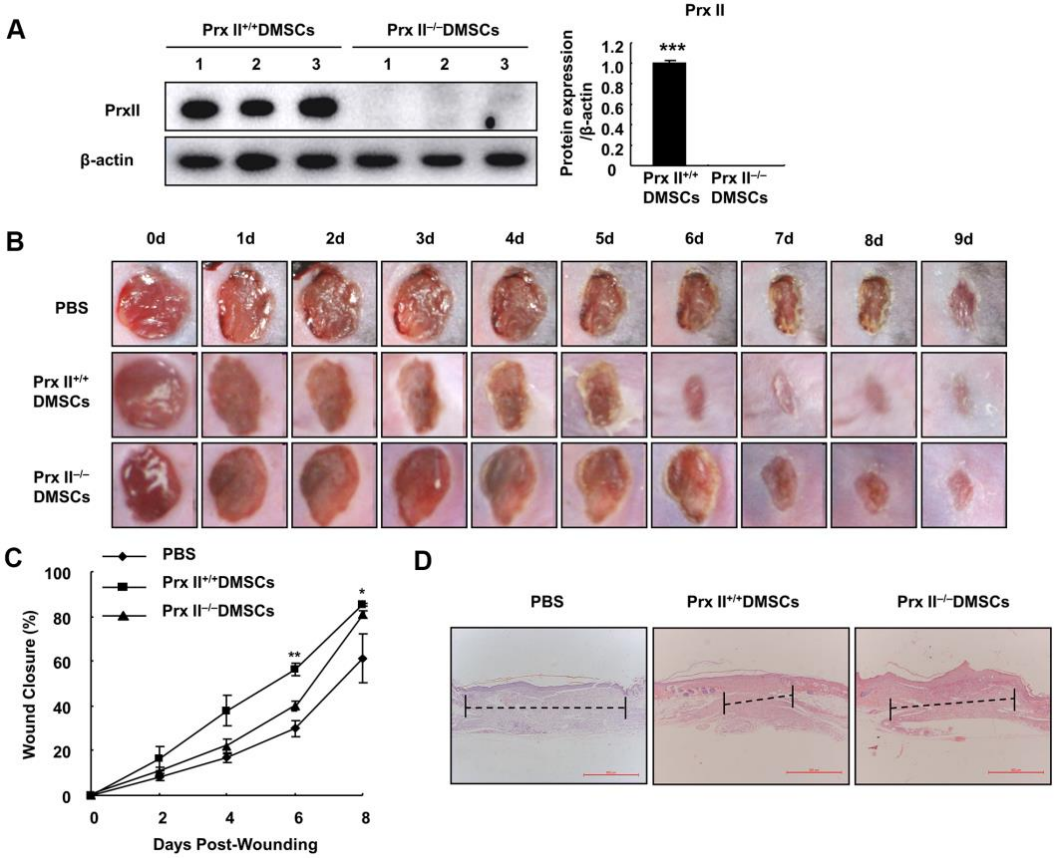
**Prx II^−/−^ DMSCs showed less skin wound healing than Prx II^+/+^ DMSCs.** (**A**) Prx II protein-expression levels in Prx II^+/+^ and Prx II^−/−^ DMSCs. (**B**) Overall observed morphological changes in wound healing after treatment. (**C**) Wound-area changes observed during wound healing. *p < 0.05, ** *p* < 0.01, when compared with Prx II^−/−^ DMSCs. The data shown represent the mean ± SD (n = 6). (**D**) Histological images (H&E staining) of wounds. Wounds are indicated with dashed imaginary lines.

To determine whether Prx II can positively regulate wound healing, we used Prx II^+/+^ DMSCs and Prx II^−/−^ DMSCs to treat full-thickness excisional cutaneous wounds in a mouse model. We detected the Prx II levels in the DMSCs used for treatment. DMSCs extracted from wild-type mice expressed normal levels of Prx II, and DMSCs extracted from Prx II-knockout mice did not express Prx II (Figure 2A). Skin wound healing was significantly accelerated in the DMSC-treated group (compared to the control group) and in the Prx II^+/+^ DMSC-treated group (compared to the Prx II^−/−^ DMSC-treated group). Assessment of wound-closure rates suggested that the Prx II^+/+^ DMSC-treated group (85.36 ± 1.25%) had significantly smaller wounds than the Prx II^−/−^ DMSC-treated group (80.76 ± 3.44%) on day 8 (Figure 2B, 2C). Furthermore, histochemical analysis of the wounds confirmed these results. Granulation tissues in Prx II^−/−^ DMSC-treated wounds appeared thicker and larger than those in Prx II^+/+^ DMSC-treated wounds (Figure 2D). These results suggest that Prx II played an important role in DMSC therapy during wound healing.

### Deletion of Prx II promoted apoptosis in DMSCs under H_2_O_2_-induced oxidative stress

**Figure 3:**
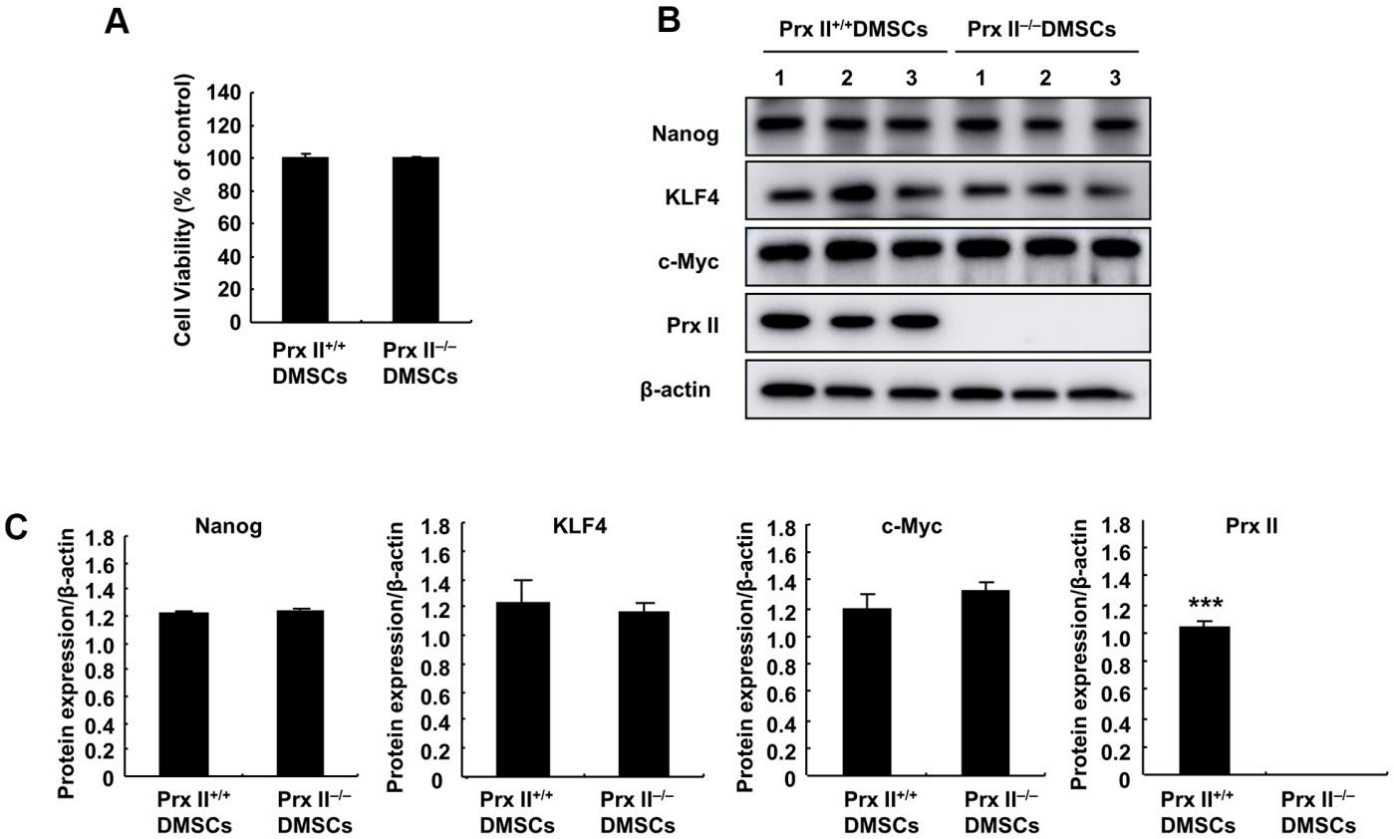
**Detection of Prx II^+/+^ DMSC and Prx II^−/−^ DMSC proliferation and stem cell stem-related proteins.** (**A**) Cell proliferation was detected by performing MTT assays after culturing for 24 h. (**B**, **C**) Western blot analysis of Prx II^+/+^ DMSC and Prx II^−/−^ DMSC extracts, and data quantification, in order to investigate stem cell stem-related proteins, such as Nanog, KLF4, and c-Myc.

**Figure 4:**
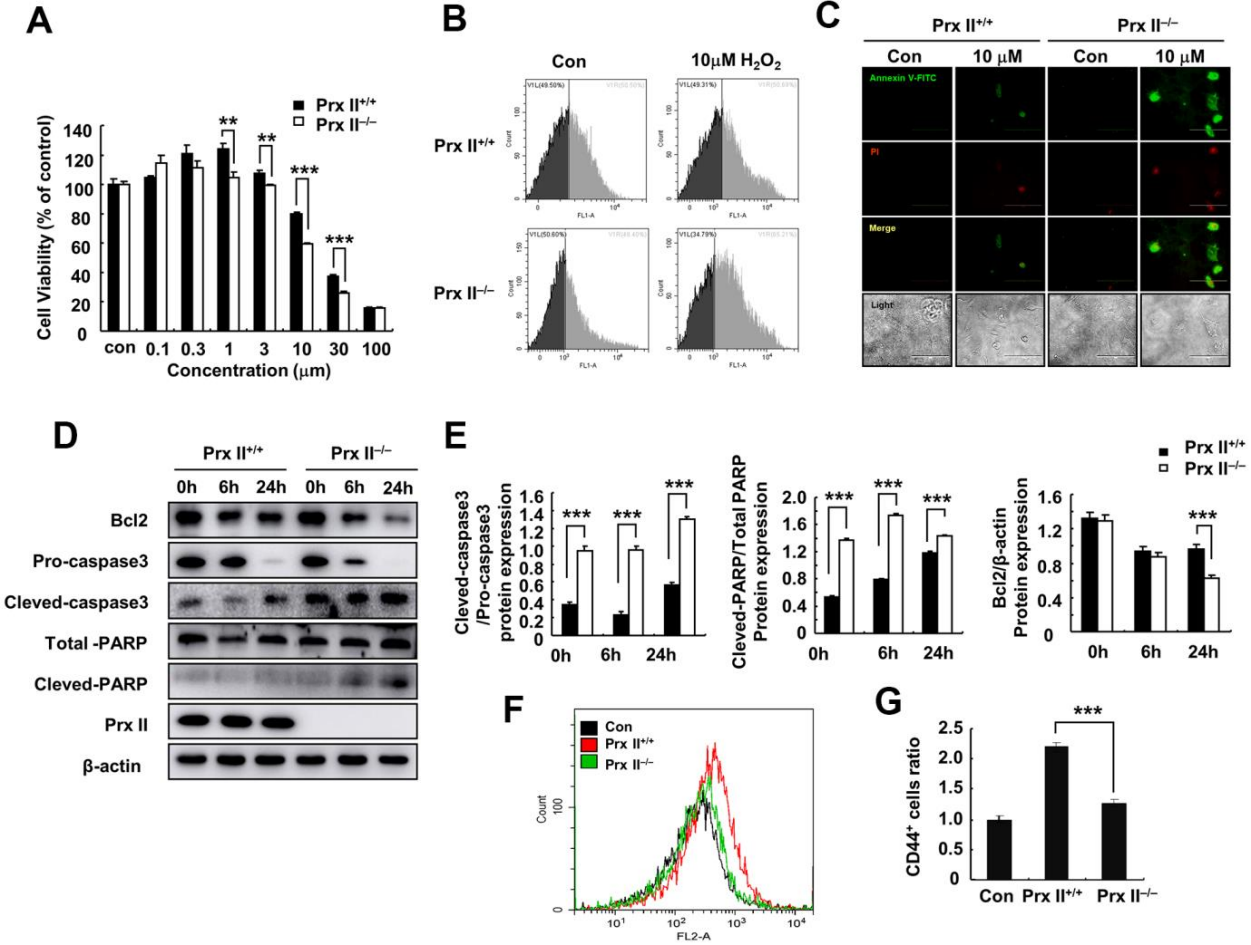
**Deletion of Prx II promoted DMSC apoptosis under H_2_O_2_-induced oxidative stress.** (**A**) Cell viabilities of Prx II^+/+^ DMSCs and Prx II^−/−^ DMSCs after treatment with increasing concentrations of H_2_O_2_. **p < 0.01, ***p < 0.001, when compared with the control group. (**B**) Cell death was detected by flow cytometry after treatment for 24 h with 10 μM H_2_O_2_. (**C**) Annexin V and PI staining were performed to visualize apoptosis after treatment for 24 h with 10 μM H_2_O_2_. (**D**, **E**) Western blotting of Prx II^+/+^ DMSC and Prx II^−/−^ DMSC extracts, and data quantification, in order to investigate the effect of 10 μM H_2_O_2_ on the expression of Prx II and apoptosis-related proteins, such as Bcl2, pro-caspase 3, and cleaved-caspase 3, total PARP, and cleaved PARP after 6 and 24 h. (**F**, **G**) Flow cytometry was used to detect the number of CD44-positive cells in the wound site after treatment with Prx II^+/+^ DMSCs and Prx II^−/−^ DMSCs treatment, and to quantify the data.

To explore the reasons for differences in wound healing, we first assessed the cell proliferation and differentiation potentials of Prx II^+/+^ DMSCs and Prx II^−/−^ DMSCs. We observed no significant difference between either parameter in Prx II^+/+^ DMSCs and Prx II^−/−^ DMSCs (Figure 3A–3C). The antioxidant potential of stem cells can affect the therapeutic potential of stem cells. Therefore, we used H_2_O_2_ to stimulate Prx II^+/+^ DMSCs and Prx II^−/−^ DMSCs *in vitro*. Prx II^−/−^ DMSCs showed lower viability than Prx II^+/+^ DMSCs, and flow cytometric analysis revealed that significantly more Prx II^−/−^ DMSCs died after H_2_O_2_ treatment *in vitro* than Prx II^+/+^ DMSCs (Figure 4A, 4B). To determine the rate of DMSC apoptosis following H_2_O_2_ treatment, we obtained fluorescence microscopy images of cells stained with fluorescein isothiocyanate (FITC)-conjugated Annexin V and propidium iodide (PI) after H_2_O_2_ treatment, and analyzed the expression levels of apoptotic proteins via western blotting. Treatment with 10 μM H_2_O_2_ induced Annexin V expression, downregulated Bcl2 expression, and upregulated cleaved caspase 3, pro-caspase 3, cleaved PARP, and total PARP. In addition, compared with Prx II^+/+^ DMSCs, H_2_O_2_ induced significantly higher levels of apoptosis in Prx II^−/−^ DMSCs (Figure 4C–4E). Furthermore, significantly less CD44-positive cells were observed at wound sites in the Prx II^−/−^ DMSC-treated group compared with the Prx II^+/+^ DMSC-treated group, as determined by flow cytometry (Figure 4F, 4G). These results indicate that Prx II deletion weakened the anti-oxidative stress capacity of DMSCs and increased apoptosis in DMSCs, leading to fewer surviving stem cells at wound sites.

### Deletion of Prx II did not influence the effect of DMSC-CM treatment on skin wound healing

**Figure 5:**
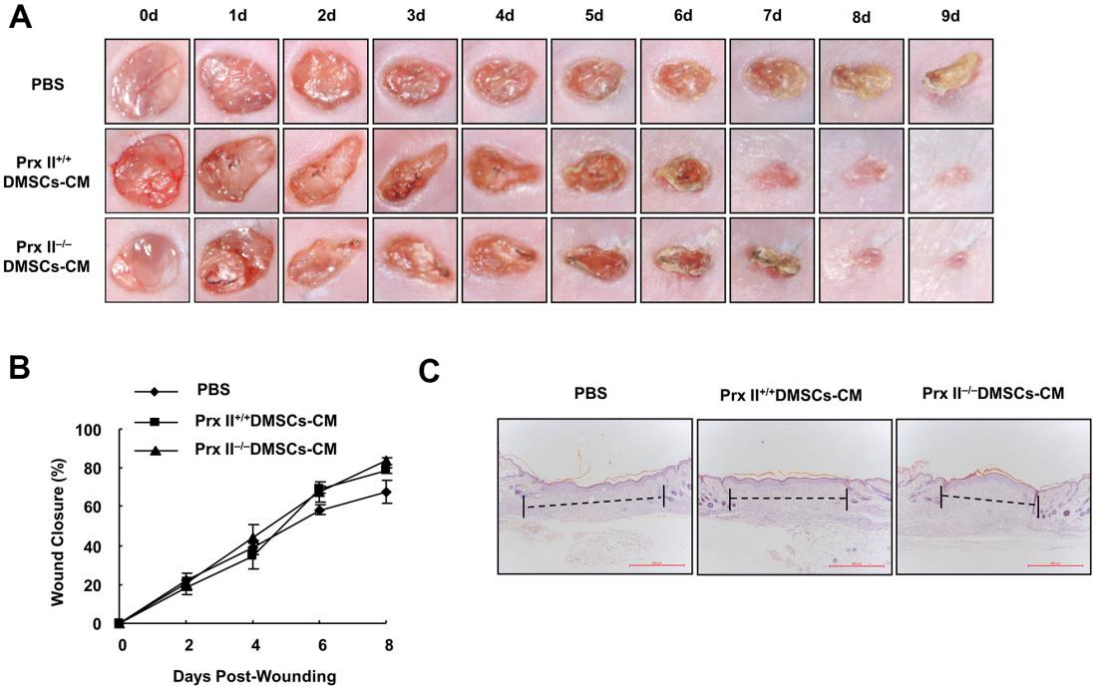
**Prx II^+/+^ DMSC-CM and Prx II^−/−^DMSC-CM promoted skin wound healing.** (**A**) Overall morphological changes observed during wound healing after treatment. (**B**) Wound-area changes observed during wound healing *p < 0.05, **p < 0.01, when compared with Prx II^−/−^ DMSC-CM. The data shown represent the mean ± SD (n = 6). (**C**) Histological images (H&E staining) of wounds. Wounds are indicated with dashed lines.

Stem cells promote wound healing, not only through proliferation and differentiation, but also through cell-growth factor and exosome secretion. During treatment, Prx II^−/−^ DMSCs showed increased apoptosis and a decreased number of cells capable of secreting cytokines and exosomes. Therefore, we attempted to evaluate the role of Prx II in DMSC-based skin wound treatment more comprehensively. Prx II^+/+^ DMSCs-CM and Prx II^−/−^ DMSCs-CM were prepared, and a mouse model of full-thickness skin wound healing was used. Prx II^+/+^ DMSCs-CM and Prx II^−/−^ DMSCs-CM significantly accelerated skin wound healing compared to phosphate-buffered saline (PBS). However, no significant difference was observed between the two groups. Furthermore, their wound-closure rates were similar. The wound-closure rate of the Prx II^+/+^ DMSC-CM-treated group (78.39 ± 2.99%) was not significantly different from that of the Prx II^−/−^ DMSC-CM-treated group (83.77 ± 3.79%) on day 8 (Figure 5A, 5B). In addition, histochemical analysis of wound tissues confirmed these results (Figure 5C). These results suggest that Prx II deletion did not influence the efficacy of DMSC-CM in promoting skin wound healing.

### Prx II did not regulate cell-growth factor secretion from DMSCs

**Figure 6:**
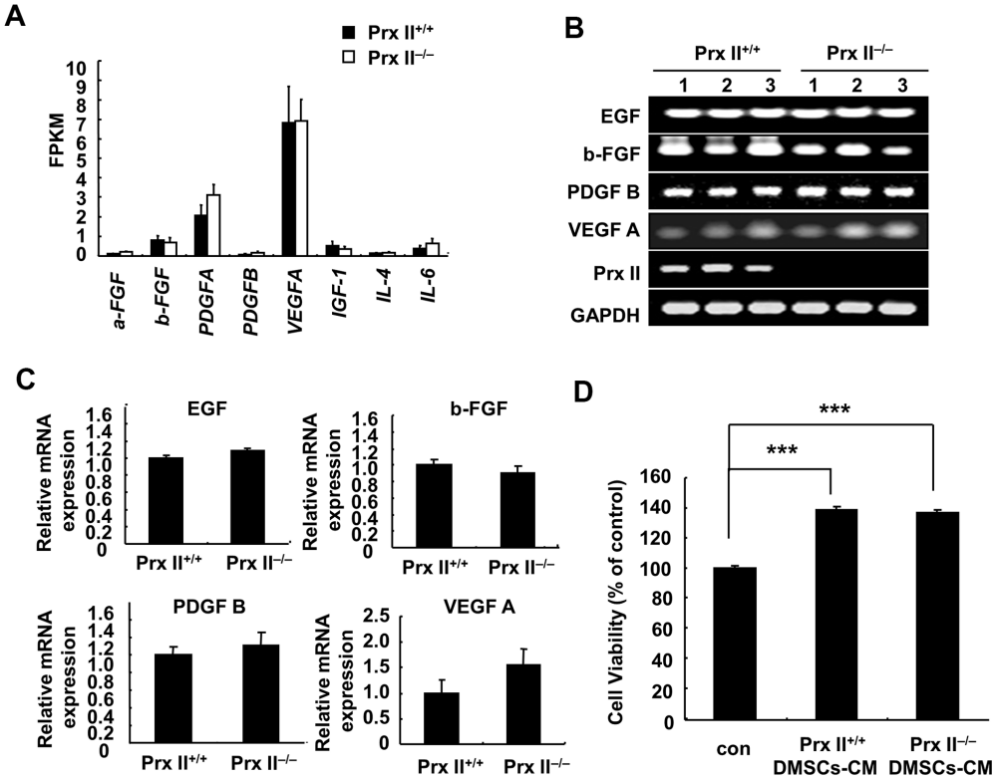
**Expression of cell-growth factors in DMSCs.** (**A**) Fragments per kilobase of transcript per million mapped reads values obtained by RNA-sequencing analysis. (**B**, **C**) Relative expression levels of four genes in DMSCs with and without Prx II expression, as determined by RT-PCR, are shown. (**D**) Proliferation of dermal fibroblasts after treatment with Prx II^+/+^ DMSC-CM or Prx II^−/−^ DMSC-CM. ***p < 0.001, when compared with the control group.

The conditioned culture medium of stem cells is rich in various growth factors that can promote wound healing [14]. Several reports have shown that the active components of MSC-CM include EGF, b-FGF, PDGF B, and VEGF A (among other factors) and that these cell-growth factors promote skin fibroblast proliferation and then enhance skin wound healing [15]. Therefore, we investigated whether Prx II can regulate cell-growth factor secretion by DMSCs. mRNA sequencing was performed to detect the mRNA levels of various cell-growth factors (Figure 6A), and reverse transcription-polymerase chain reaction (RT-PCR) analysis was performed to detect the mRNA levels of EGF, b-FGF, PDGF-B, and VEGF-A (which had pro-proliferative effects on fibroblasts) in Prx II^+/+^ and Prx II^−/−^ DMSC-CM. Statistical analysis revealed no significant differences in growth factors in Prx II^+/+^ DMSCs and Prx II^−/−^ DMSCs (Figure 6B, 6C). To confirm the effect of DMSC-CM-induced proliferation in fibroblasts, we measured the proliferation of primary dermal fibroblasts treated with Prx II^+/+^ DMSC-CM or Prx II^−/−^ DMSC-CM. DMSC-CM significantly promoted dermal fibroblast proliferation, but no difference was observed in the promotion effects between Prx II^+/+^ DMSC-CM and Prx II^−/−^ DMSC-CM (Figure 6D). These findings are consistent with the results of our *in vivo* experiments on DMSC-CM treatment. Our results suggest that Prx II does not regulate cell-growth factor secretion by DMSCs, leading to the same pro-proliferation effect on dermal fibroblasts during the skin wound-healing process. Therefore, no significant difference was observed between Prx II^+/+^ DMSC-CM and Prx II^−/−^ DMSC-CM during the treatment of skin wounds.

### Characterization of DMSC-Exos

**Figure 7:**
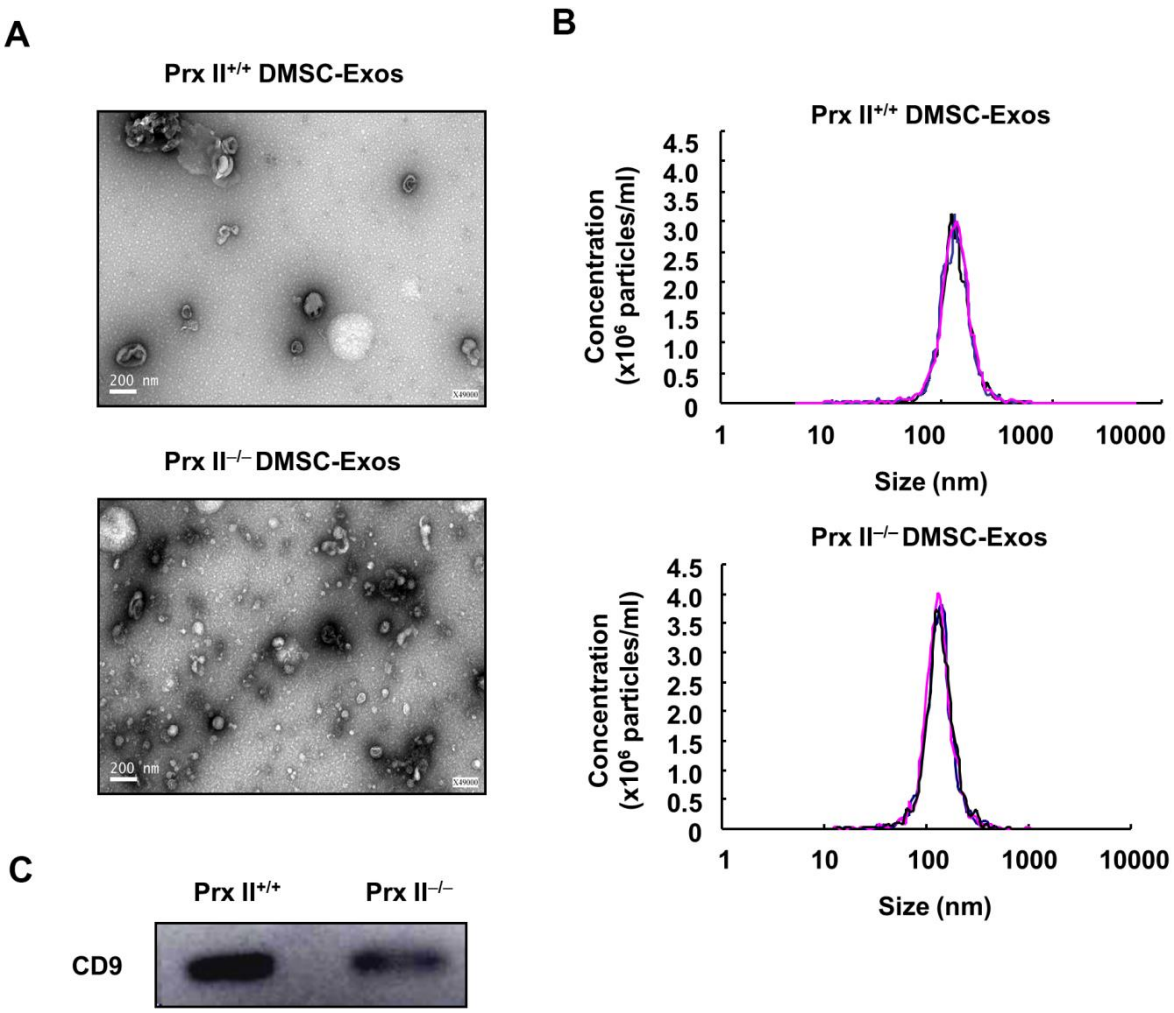
**Extraction and identification of Prx II^+/+^ DMSC-Exos and Prx II^−/−^ DMSC-Exos.** (**A**) Morphological observations of exosomes by electron microscopy. (**B**) Particle-size analysis of exosomes based on flow cytometry. (**C**) Western blot analysis of exosomal extracts to investigate the expression of surface markers.

Exosomes are vesicles with diameters ranging from 40 nm to 200 nm that can be released into the extracellular environment [16]. Data from numerous animal studies have shown that MSC exosomes regulate inflammation, cell proliferation, migration, angiogenesis, and matrix reconstruction in wound healing [17, 18]. To verify the role of Prx II in the treatment of skin wound healing using exosomes derived from DMSCs, Prx II^+/+^ DMSC-Exos and Prx II^−/−^ DMSC-Exos were extracted, and their ultrastructures and particle-size distributions were analyzed through transmission electron microscopy and nanoparticle-tracking analysis. The vesicles had a characteristic cup-shaped morphology (Figure 7A) and the size distribution of most exosomes in both groups ranged from 40 nm to 200 nm (Figure 7B). Western blotting was performed to analyze expression of the exosomal surface marker, CD9 (Figure 7C).

### Prx II deletion promoted DMSC-Exo-based skin wound healing

**Figure 8:**
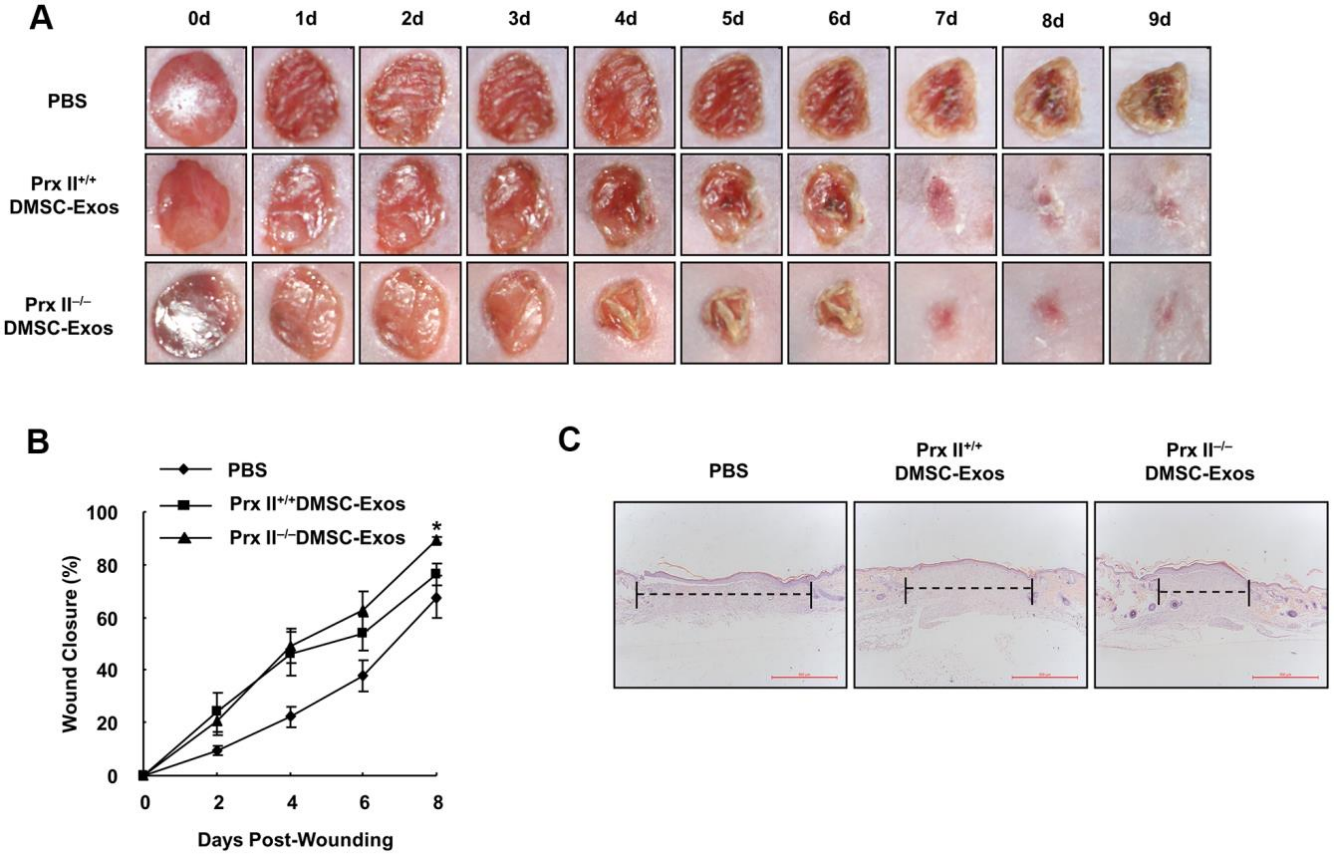
**Prx II^−/−^DMSC-Exos promoted skin wound healing more effectively than Prx II^+/+^ DMSC-Exos.** (**A**) Overall observed morphological changes in wound healing after treatment. (**B**) Wound-area changes during wound healing are shown. *p < 0.05, when compared with Prx II^−/−^ DMSC-Exos. The data shown represent the mean ± SD (n = 6). (**C**) Histological images (H&E staining) of the wound. Wounds are indicated with dashed lines.

Subsequently, we comprehensively evaluated the role of Prx II in DMSCs used to treat skin wounds. Prx II^+/+^ DMSC-Exos and Prx II^−/−^ DMSC-Exos were prepared, and a mouse model of full-thickness skin wound healing was used. We found that the DMSC-Exo-treated group significantly accelerated skin wound healing compared with the control group. Furthermore, the Prx II^−/−^ DMSC-Exo-treated group (89.60 ± 3.89%) had significantly smaller wounds than the Prx II^+/+^ DMSC-Exo-treated group (74.02 ± 8.86%) at day 8 (Figure 8A, 8B). In addition, histochemical analysis of wound tissues confirmed these results (Figure 8C). These results suggest that Prx II deletion affected skin wound healing by regulating exosome secretion by DMSCs.

### Prx II may regulate miR21-5p and miR221 in DMSC-Exos

**Figure 9:**
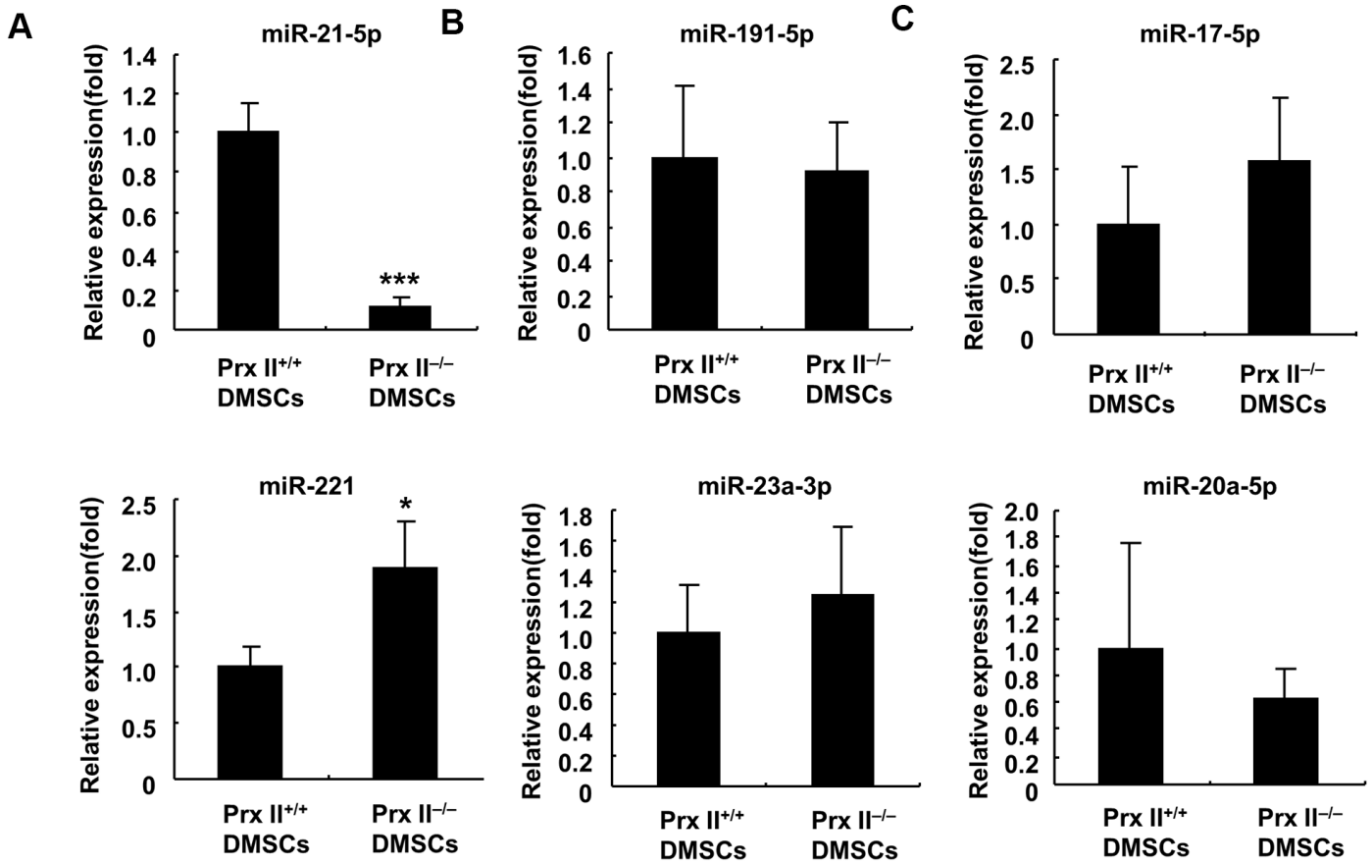
**Analysis of miRNA expression in Prx II^+/+^ DMSCs and Prx II^−/−^ DMSCs by RT-PCR.** (**A**) Expression levels of miR-21-5p and miR-221. (**B**) Expression levels of miR-23a-3p and miR-191-5p. (**C**) Expression levels of miR-20a-5p and miR-17-5p. All data shown represent the mean ± SD (n = 6).

DMSC-Exos primarily function through microRNAs (miRNAs) during skin wound healing [19, 20]. Therefore, we studied the expression of six miRNAs in Prx II^+/+^ DMSCs and Prx II^−/−^DMSCs. Quantitative PCR revealed that miR-221 expression was significantly higher in Prx II^−/−^ DMSCs than in Prx II^+/+^ DMSCs. miR-21-5p was significantly downregulated (Figure 9A), and miR-23a-3p, miR191-5p, miR-20a-5p, and miR-17-5p displayed no significant expression differences between Prx II^+/+^ DMSCs and Prx II^−/−^ DMSCs (Figure 9B, 9C).

miRNA can be selectively encapsulated into exosomes when exosomes are formed within cells. Furthermore, miR-221 and miR-21-5p are also highly expressed in miRNAs in MSCs [20]. Therefore, we believe that miR-221 was significantly upregulated in Prx II^−/−^ DMSCs due to decreased miR-221 sorting into exosomes. Similarly, miR-21-5p was downregulated in Prx II^−/−^ DMSCs due to increased miR-21-5p sorting into exosomes. Therefore, we speculate that miR-221 downregulation and miR-21-5p upregulation in Prx II^−/−^ DMSC-Exos can promote skin wound healing, owing to the negative role of miR-221 in skin wound healing and the positive role of miR-21-5p.

## DISCUSSION

During skin inflammation, ROS are produced at higher concentrations in the wound microenvironment [21]. Transplanted stem cells may be stimulated by ROS, affecting the function of stem cells and ultimately leading to a reduction in wound healing. Previous data showed that treatment with a combination of stem cells and the antioxidant, vitamin C, had a better therapeutic effect on wound healing [22, 23]. Treating wounds with H_2_O_2_-pretreated stem cells was significantly more effective, versus untreated stem cells [5], probably because vitamin C or H_2_O_2_ pretreatment increased antioxidant stress in stem cells. Therefore, because we found that the therapeutic effect of wild-type DMSCs was significantly higher than that of Prx II-knockout DMSCs, we hypothesize that the absence of Prx II affects the resistance of DMSCs to ROS and increases apoptosis. Further, we treated two types of DMSCs with H_2_O_2_; the ability of DMSCs to resist ROS decreased and apoptosis increased when Prx II was absent. Furthermore, our *in vivo* experiments showed that the number of stem cells at the wound site was reduced in the Prx II^−/−^ DMSC-treated group, consistent with previous findings. Thus, the primary explanations for the reduced therapeutic effect of Prx II-knockout DMSCs include a decreased ability to resist antioxidant stress, increased apoptosis, and the number of stem cells with a reduced proliferation and differentiation potential. Prx II has been suggested to directly protect DMSCs during skin wound treatment and to inhibit cell damage caused by ROS, thereby enhancing the therapeutic effect.

Numerous reports have shown that conditioned medium from stem cells can promote skin wound healing, owing to the presence of cell-growth factors [14, 24]. The expression levels of cell-growth factors can directly determine the efficiency of skin wound healing. For instance, VEGF and b-FGF levels in adipose MSCs cultured in a low-oxygen environment were significantly increased, as were the migration abilities of dermal fibroblasts [25]. The wound-healing rate of conditioned medium was accelerated and TGF-β3 was overexpressed in BMSCs, which significantly improved wound repair and reduced scar tissue formation in animal models [26]. Our results indicated that no significant difference occurred in wound healing stimulated by either type of DMSC-CM, and we detected the mRNA levels of cell-growth factors in DMSC-CM for the first time. No significant changes in their expression levels were observed after Prx I was knocked out, in agreement with our previous conclusion. Further, we treated fibroblasts with Prx II^+/+^ DMSC-CM and Prx II^−/−^ DMSC-CM. Fibroblasts can form granulation tissue during skin wound healing and are important target cells for cell-growth factors. Furthermore, we found that although Prx II^+/+^ DMSC-CM and Prx II^−/−^ DMSC-CM significantly promoted fibroblast proliferation during wound healing, and no significant difference was observed when compared with the control group. These results indicate that Prx II did not regulate the expression of cellular growth factors when treating skin wounds using DMSCs.

Stem cell exosomes are biologically active substances secreted by stem cells. Recent reports have shown that stem cells elicit a significant effect on skin wound healing [27]. In a rat model of deep second-degree burn wounds, MSC-Exos promoted the regeneration of epidermis and dermis cells and angiogenesis to accelerate wound healing [28]. MSCs-Exo can improve the wound-closure and re-epithelialization rates; reduce scar width; and improve collagen maturity, sebaceous gland and hair follicle formation, neovascularization, and mature vascular density [29]. However, the components of exosomes are complex. miRNAs play a major role in exosome function [30]. miR-21 plays a positive regulatory role in wound healing. In the inflammatory-response stage, miR-21 can prevent inflammation by targeting PDCD4 and can promote cell proliferation and survival by activating the mTOR pathway. Moreover, miR-21 can promote keratinocyte migration and epithelial reconstruction [31, 32]. In contrast, miR-221 plays a negative regulatory role in wound healing and can downregulate nitric oxide, inhibit vascular tubule formation by endothelial cells, and reduce the migration ability [20, 33]. Therefore, we conclude that Prx II deletion decreased miR-21-5p levels (a positive effect) and increased miR-221 levels (an inhibitory effect) in Prx II^−/−^ DMSCs. Interestingly, however, Prx II^−/−^ DMSC-Exos showed better wound healing capacity. This evidence suggests that Prx II deletion may lead to miR-21-5p accumulation in exosomes, or its exporting and capsuling, and the intracellular retention of miR-221. Moreover, similar to exosome therapy, transferring mitochondria from healthy stem cells to cells with damaged mitochondria can restore their aerobic respiratory function and, thus, accentuate the therapeutic roles of stem cells [33]. These data suggest prospects for developing stem cell therapy.

**Figure 10:**
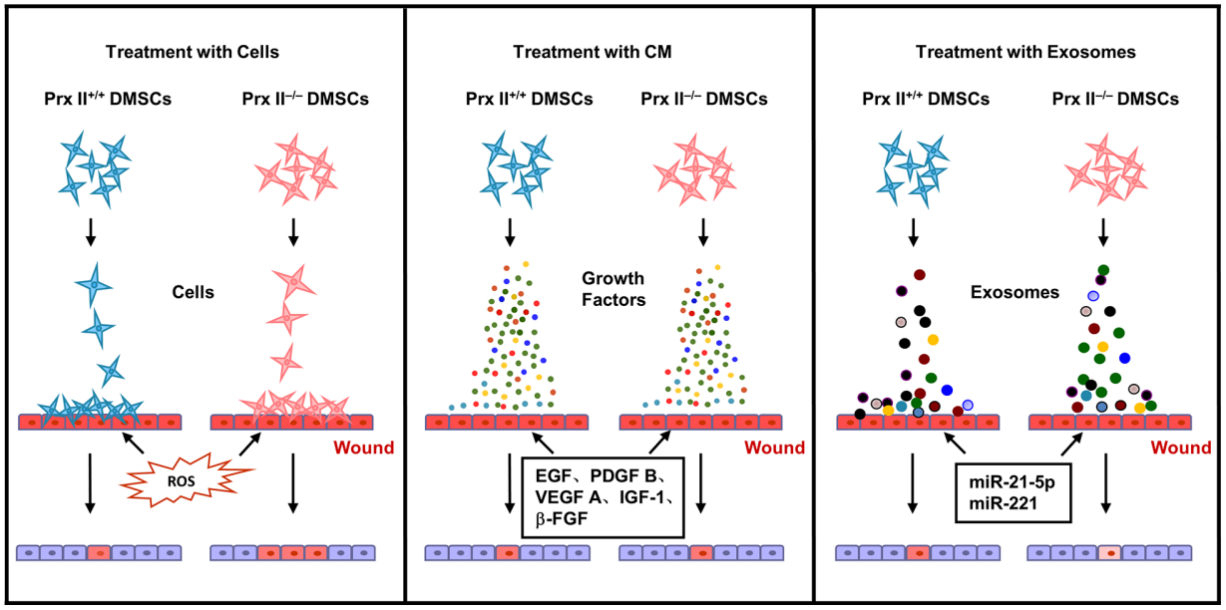
Proposed mechanism whereby Prx II regulates wound healing in DMSCs.

In conclusion, stem cell-based treatment of skin wounds is a very complicated biological phenomenon, and the modification of Prx II gene expression may change the ability of DMSCs to proliferate, differentiate, or secrete biologically active substances. These changes are not necessarily beneficial in skin wound healing, and it is important to explore the role of Prx II comprehensively and systematically, as well as the regulatory mechanism of Prx II when treating skin wounds with DMSCs, in order to determine the optimal treatment method in subsequent clinical applications (Figure 10).

## MATERIALS AND METHODS

### Ethics statement

The Institutional Animal Ethic Committee (TDJH201916, Heilongjiang Bayi Agricultural University, Daqing, China) approved both the animal care and experimental protocols.

### Isolation of DMSCs and DMSC-Exos, and preparing DMSC-CM

DMSCs were isolated as described previously [8]. Briefly, wild-type and Prx II-knockout 129/SvJ mice (Korea Research Institute of Bioscience and Biotechnology) were used. The skin surface of the mice was disinfected with 70% ethanol after anesthesia with ethyl ether. Finally, the dorsal skin was dissected. The skin samples were digested in 0.25% trypsin-EDTA (Solarbio® Life Sciences, Beijing, China) and seeded in Dulbecco’s modified Eagle’s medium (DMEM)/F12 medium (Gibco BRL, Grand Island, NY, USA). At passage four, the DMSCs were immunostained for 30 min at 4° C with fluorochrome-conjugated antibodies, such as anti-CD44-PE, anti-CD106-PE, anti-CD14-FITC, anti-CD34-PE, and anti-CD45-FITC (all from BioLegend; San Diego, CA, USA). DMSC phenotypes were analyzed through flow cytometric analysis (BD Biosciences, San Jose, CA, USA). DMSCs were separately cultured in osteogenic differentiation medium (Solarbio® Life Sciences) and lipogenic differentiation medium (Solarbio® Life Sciences). After 21 days, the cells were stained using alizarin red and oil red O (Solarbio® Life Sciences). Ultimately, DMSCs were imaged using a fluorescence microscope coupled with a camera (Leica DM2500, Leica, Wetzlar, Germany).

DMSCs were seeded in 10 mm^2^ tissue-culture flasks with fresh medium. When the cell density approached 80%, fresh medium containing 10% fetal bovine serum and lacking exosomes (eliminated through ultracentrifugation for 16 h at 120,000 × *g*, 4° C) was added, and supernatants were obtained after 24 h. Exosomes were extracted through high-speed centrifugation, as described previously [34]. The final pellets from 100 mL supernatants were resuspended in 200 μL PBS and stored at -80° C. Ultrastructures and particle-size distributions were analyzed by transmission electron microscopy with Nanoparticle Tracking Analysis software, version 2.2 (XP Biomed, Shanghai, China).

To obtain DMSC-CM, DMSCs at passage four were cultured to 80% confluence in serum-free DMEM. Negative-control medium was obtained under the same culture conditions, but without cells. After 12 h, the conditioned medium was harvested. The supernatant was centrifuged at 300 × *g* for 10 min and filtered through a 0.22 μm syringe filter. For *in vivo* experiments, the DMSC-CM was further concentrated to 5× using a freeze-drying machine. To generate a carbomer gel, carbomer were added to double-distilled water, NaOH was added under aseptic conditions, the mixture was allowed to stand for 12 h, DMSC-CM was added, and the resulting gel was stored at 4° C until use.

### Skin-wound modeling and treatment

Wild-type 129/SvJ mice (12–16 weeks old; body weight, 20–23 g) were obtained from the Korea Research Institute of Bioscience and Biotechnology (KRIBB). The animals were randomly divided into groups, and wound healing was studied as described previously [35]. Briefly, the mice were anesthetized with 0.25% avertin through intraperitoneal injection at a dose of 250 mg/kg. Thereafter, iodine and 70% alcohol were used to disinfect the skin. Furthermore, hair was removed from the dorsal surface. Two full-thickness excisional wounds with a 5 mm diameter were inflicted on each side. DMSC treatment: after 4 h, 2 × 10^6^ DMSCs (in 200 μL PBS) was injected intradermally around the wound at four injection sites. An equal amount of PBS was injected into the control mice. DMSC-CM treatment: skin-wound model mice were treated with 50 μL DMSC-CM hydrogel, applied to the wound bed every day; an equal amount of carbomer hydrogel gel was used as a negative control. DMSC-Exo treatment: skin-wound model mice were treated with 8 μg DMSC-Exos, which was injected subcutaneously around the wound at four sites. An equal amount of PBS was injected in the same manner into the control mice. The initial wound sizes were similar between the groups.

### Wound-repair analysis

Digital photographs of the excisional wounds were obtained on days 0–9. The wound area was measured by tracing the wound margin and calculated using the ImageJ analysis program (https://imagej.nih.gov/ij/index.html, National Institutes of Health, Bethesda, MD, USA). The percentage of wound closure was calculated as follows: wound closure percentage = ([area of original wound − area of actual wound]/area of original wound) × 100. The mice were anesthetized and the dorsal skin was removed on day 9 after wound surgery. Each sample was cut and placed in buffered formalin solution for histopathological examination. Tissue sections were stained with hematoxylin and eosin (H&E) and examined by pathologists.

### Cell-viability assay

Cell viability was evaluated by performing 3-(4,5-dimethylthiazol-2-yl)-2,5-diphenyltetrazolium bromide (MTT) assays. DMSCs were seeded at a density of 20,000 cells per well in 48-well plates and treated with 0.1–100 μM H_2_O_2_ for 24 h (37° C and 5% CO_2_). Thereafter, the MTT reagent was added to the wells in each plate (final concentration, 5 mg/mL), followed by 200 μL of dimethyl sulfoxide after 4 h, after which the absorbance was measured at 490 nm. Absorbance was detected using a Microplate Absorbance Reader (Molecular Devices, LLC, Sunnyvale, CA, USA).

### Immunofluorescence staining

DMSCs were seeded at a density of 40,000 cells per well in 24-well plates and treated with 10 μM of H_2_O_2_ for 24 h (37° C and 5% CO_2_). The medium was removed and the cells were stained with Annexin V-FITC and PI using an apoptosis detection kit (BD Biosciences, Franklin Lakes, NJ, USA) in accordance with the manufacturer’s protocol. Finally, a Leica DM2500 fluorescence microscope was used for imaging.

### Western blotting

For each sample, total protein was separated by performing sodium dodecyl sulfate-polyacrylamide gel electrophoresis (15% gel), and the proteins were electro-transferred onto nitrocellulose membranes (Millipore, Bedford, MA, USA). The membranes were washed five times with Tris-buffered saline. The following primary antibodies were used: anti-Prx II (AbFrontier, Seoul, Republic of Korea), anti-cleaved PARP (Santa Cruz Biotechnology, Santa Cruz, CA, USA), anti-total-PARP (Elabscience Biotechnology, Wuhan, China), anti-pro-caspase 3 (Cell Signaling Technology, CA, USA), anti-cleaved-caspase 3 (Santa Cruz Biotechnology), anti-Bcl2 (Santa Cruz Biotechnology), anti-CD9 (Solarbio® Life Sciences, Beijing, China), and anti-β-actin (Santa Cruz Biotechnology). As secondary antibodies, we used a goat anti-mouse antibody (ZSGB-BIO, Beijing, China) and a goat anti-rabbit antibody (ZSGB-BIO, Beijing, China). The blots were imaged with Alpha View Software (AlphaView, USA) and analyzed using ImageJ software.

### Cytokine assay

To analyze factors secreted by DMSCs, RNA was extracted using the TRIzol® reagent (Sigma, St. Louis, MO, USA) and analyzed through mRNA sequencing using a HiSeq instrument (Genminix, Shanghai, China). Prx II^+/+^ and Prx II^−/−^ DMSCs were each analyzed in triplicate.

Total cellular RNA was prepared using the TRIzol® reagent (Invitrogen, Carlsbad, CA, USA), followed by complementary DNA (cDNA) synthesis using Reverse Transcriptase II (Invitrogen), in accordance with the manufacturer’s instructions. The cDNA was amplified using the following PCR primers: EGF (5′-ACACGGAGGGAGGCTACA-3′ and 5′-GTAGCCTCCCTCCGTGTT-3′), b-FGF (5′-AGTCTTCGCCAGGTCATTGA-3′ and 5′-CCTGAGTATTCGGCAACAGC-3′), PDGF-B (5′-GATCCGCTCCTTTGATGATC-3′ and 5′-GTCTCACACTTGCATGCCAG-3′), VEGF-A (5′-CTTCTGAGTTGCCCAGGAGA-3′ and 5′-CTCACACACACACAACCAGG-3′), PRX II (5′-AGGACTTCCGAAAGCTAGGC-3′ and 5′-GGTTGCTGTCATCCACATTG-3′), and GAPDH (5′-TGTGTCCGTCGTGGATCTGA-3′ and 5′-CCTGCTTCACCACCTTCTTGA-3′). Thermocycling was performed using an initial 94° C hold step for 5 min. This hold step was followed by 25–30 cycles of 94° C for 30 s; 58° C, 54° C, or 52° C for 30 s; and 72° C for 30 s; and a final extension step for 5 min at 72° C. The amplified samples were electrophoresed on 1% agarose gels and quantified using Alpha View Software (AlphaView).

### Measurement of dermal fibroblast proliferation

Cell growth was assessed by plating dermal fibroblasts at a density of 10^4^ cells/well in 48-well plates. After 24 h, the medium was removed, and the cells were washed twice with PBS and treated with 200 μL of DMSC-CM or non-conditioned medium for 24 h. Thereafter, the MTT reagent was added to each well at a final concentration of 5 mg/mL, followed by 200 μL dimethyl sulfoxide. After 4 h, the absorbance was measured at 490 nm using a Microplate Absorbance Reader (Molecular Devices, LLC, Sunnyvale, CA, USA). The experiments were performed in triplicate.

### Quantitative real-Time PCR

Total cellular RNA was prepared with TRIzol^®^ reagent (Invitrogen). miRNA expression was examined using the miRNA First Strand cDNA Synthesis kit (Tailing Reaction) (Sangon Biotech, Shanghai, China) in accordance with the manufacturer’s instructions. Real-Time PCR was performed using a QuantStudio Dx Real-Time PCR Instrument (Thermo Fisher, Waltham, MA, USA), using the following primers: miR-191-5p (5′-CAACGGAATCCCAAAAGCAG-3′ and 5′-CCAGTGAGCAGAGTGACG-3′), miR-23a-3p (5′-CCAGGAACCCCTCCTTACTC-3′ and 5′-TCTAGGGATGGTCCGAAGGA-3′), miR-17-5p (5′-TGGGCAAAGTGCTTACAGTG-3′ and 5′-CAGTGCGTGTCGTGGAGT-3′), miR-199a-5p (5′-GGCGCCCAGTGTTCAGACTAC-3′ and 5′-GTGCAGGGTCCGAGGT-3′), miR-205 (5′-CTTGTCCTTCATTCCACCGGA-3′ and 5′- TGCCGCCTGAACTTCACTCC-3′), miR-221 (5′-GGGAAGCTACATTGTCTGC-3′ and 5′-CGRTGCGTGTCGTGGAGT-3′), miR-20a-5p (5′-TCGGGTAAAGTGCTTATAGTGC-3′ and 5′-CAGTGCGTGTCGTGGAGT-3′), and miR-34c-5p (5′-GCGAGTTACTAGTAGGCAGTGTAGTTAG-3′ and 5′-AGTGCGTGTCCTGCTGTCG-3′). U6 small nucleolar mRNA was detected as an internal miRNA control. The relative expression levels were evaluated using 2-DDCT values for each sample.

### Statistical analysis

All data are presented as the mean ± standard deviation (SD) from at least three independent experiments. Paired Student’s t-tests and two-way analysis of variance were performed, followed by Tukey’s post-hoc test. A P-value of <0.05 was considered to reflect a statistically significant difference. SPSS Statistics Software, version 25 (IBM) was used for all statistical analysis.
